# Supplementary material for: Cytosolic HMGB1 Mediates LPS-Induced Autophagy in Microglia by Interacting with NOD2 and Suppresses Its Proinflammatory Function
Source: Cells. 2022 Aug 4;11(15):2410. doi: 10.3390/cells11152410 (PMC9368039; doi:10.3390/cells11152410)
Supplement: Supplementary file 1 [file cells-11-02410-s001.zip › cells-1807154-supplementary.pdf]

Supplementary materials

Cytosolic HMGB1 mediates LPS-induced autophagy in microglia by interacting with NOD2 and suppresses its proinflammatory function

Seung-Woo Kim[b,#], Sang-Ah Oh[a,#], Song-I Seol[a], Dashdulam Davaanyam[a], Ja-Kyeong Lee[a,*]

[a]Department of Anatomy, Inha University School of Medicine, Incheon, 22212, Korea
[b]Department of Biomedical Sciences, Inha University School of Medicine, Inchon 22212, Korea

[#] These authors are equally contributed

Key words: HMGB1; NOD2; Autophagy; inflammation; microglia

[*]Corresponding author:

Ja-Kyeong Lee, Ph.D.

Department of Anatomy, Inha University School of Medicine

Inharo 100, Inchon, 22202, Republic of Korea

Tel, +82-32-860-9893; jklee@inha.ac.kr

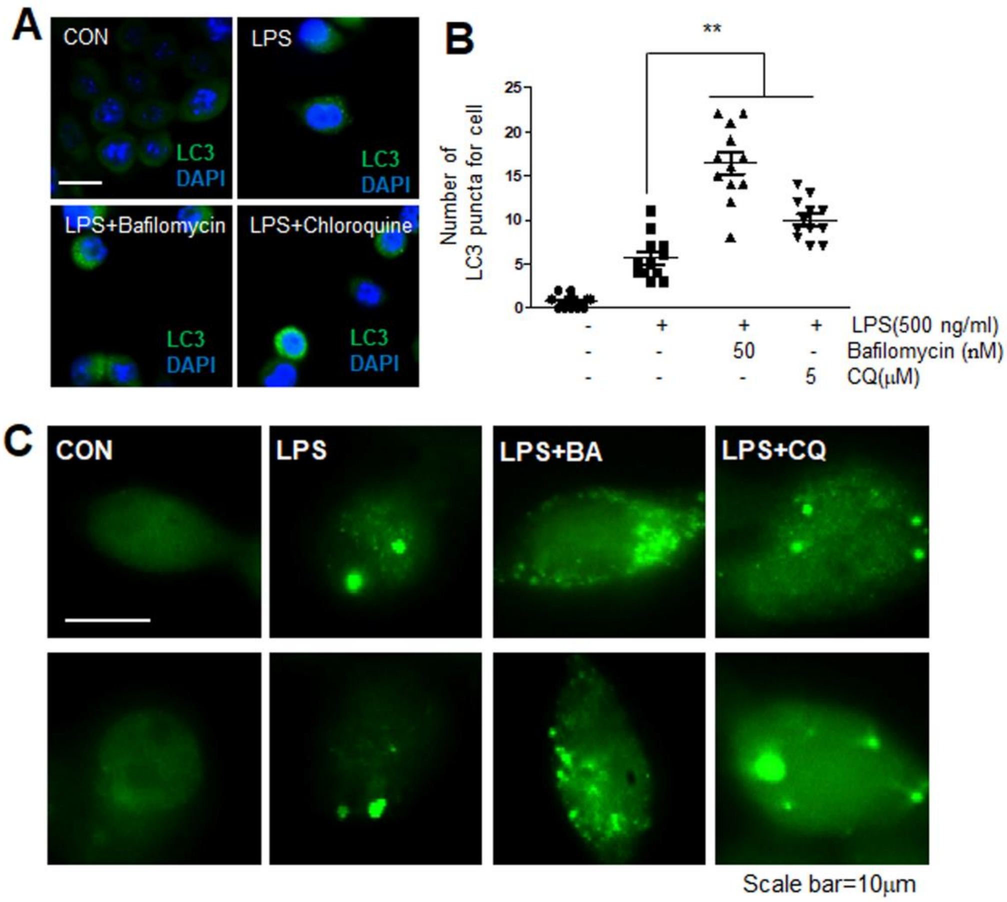

Supplementary Figure S1. Suppression of LPS-mediated autophagy flux in BV2 cell by Chloroquine and Bafilomycin A1

BV2 cells were pretreated with Bafilomycin A1 (BA, 50 nM) or chloroquine (CQ 5 mM) for 2 h and then treated with LPS (500 ng/ml) for 24 h. Immunofluorescence staining was conducted at 12 and 24 h after LPS treatment with anti-LC3 antibody (green) and DAPI (blue). Representative images are shown in A, and quantification of the dot- or ring-shaped LC3 signals (representing autophagosomes) are shown in B (n=12). Images in C are high magnification pictures. Scale bar, 20 μm for A and 10 μm for C.

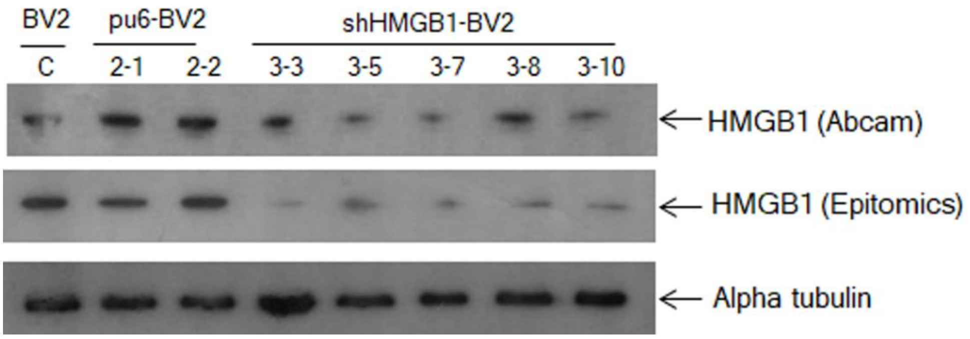

Supplementary Figure S2. Generation of HMGB1-deficient stable BV2 cell lines

BV2 cells were transfected with pU6 plasmid expressing HMGB1 shRNA (shHMGB1-BV2) or empty pU6 plasmid (pU6-BV2). At 24 h post transfection, HMGB1 expression levels were examined in five cell lines after clonal selection using two different antibodies. We selected one cell line, namely, shHMGB1-BV2 (3-5), was selected and used in the subsequent experiments.
